# An Epidemiological Model Considering Isolation to Predict COVID-19 Trends in Tokyo, Japan: Numerical Analysis

**DOI:** 10.2196/23624

**Published:** 2020-12-16

**Authors:** Motoaki Utamura, Makoto Koizumi, Seiichi Kirikami

**Affiliations:** 1 Research Laboratory for Nuclear Reactors Tokyo Institute of Technology Tokyo Japan; 2 Hitachi Research Laboratory Hitachi Ltd Hitachi Japan; 3 Hitachi Works Hitachi Ltd Hitachi Japan

**Keywords:** coronavirus, COVID-19, epidemiological model, prediction, Tokyo, delay differential equation, SIR model, model, epidemiology, isolation, trend

## Abstract

**Background:**

COVID-19 currently poses a global public health threat. Although Tokyo, Japan, is no exception to this, it was initially affected by only a small-level epidemic. Nevertheless, medical collapse nearly happened since no predictive methods were available to assess infection counts. A standard susceptible-infectious-removed (SIR) epidemiological model has been widely used, but its applicability is limited often to the early phase of an epidemic in the case of a large collective population. A full numerical simulation of the entire period from beginning until end would be helpful for understanding COVID-19 trends in (separate) counts of inpatient and infectious cases and can also aid the preparation of hospital beds and development of quarantine strategies.

**Objective:**

This study aimed to develop an epidemiological model that considers the isolation period to simulate a comprehensive trend of the initial epidemic in Tokyo that yields separate counts of inpatient and infectious cases. It was also intended to induce important corollaries of governing equations (ie, effective reproductive number) and equations for the final count.

**Methods:**

Time-series data related to SARS-CoV-2 from February 28 to May 23, 2020, from Tokyo and antibody testing conducted by the Japanese government were adopted for this study. A novel epidemiological model based on a discrete delay differential equation (apparent time-lag model [ATLM]) was introduced. The model can predict trends in inpatient and infectious cases in the field. Various data such as daily new confirmed cases, cumulative infections, inpatients, and PCR (polymerase chain reaction) test positivity ratios were used to verify the model. This approach also derived an alternative formulation equivalent to the standard SIR model.

**Results:**

In a typical parameter setting, the present ATLM provided 20% less infectious cases in the field compared to the standard SIR model prediction owing to isolation. The basic reproductive number was inferred as 2.30 under the condition that the time lag *T* from infection to detection and isolation is 14 days. Based on this, an adequate vaccine ratio to avoid an outbreak was evaluated for 57% of the population. We assessed the date (May 23) that the government declared a rescission of the state of emergency. Taking into consideration the number of infectious cases in the field, a date of 1 week later (May 30) would have been most effective. Furthermore, simulation results with a shorter time lag of *T*=7 and a larger transmission rate of α=1.43α0 suggest that infections at large should reduce by half and inpatient numbers should be similar to those of the first wave of COVID-19.

**Conclusions:**

A novel mathematical model was proposed and examined using SARS-CoV-2 data for Tokyo. The simulation agreed with data from the beginning of the pandemic. Shortening the period from infection to hospitalization is effective against outbreaks without rigorous public health interventions and control.

## Introduction

COVID-19 currently represents a global public health threat. Tokyo, Japan, is no exception, but its epidemic was small despite lacking rigorous public health intervention. The thorough behavior changes of individuals, with social distancing and avoidance of the 3 Cs [1], that is, (1) closed spaces with poor ventilation, (2) crowded places with many people, and (3) close-contact settings such as close-range conversations, appear to explain Japan’s ability to slow the spread of SARS-CoV-2. Medical collapse, however, nearly occurred due to a lack of beds to accommodate the increasing number of patients [2]. Therefore, to cope with future epidemics, mathematical prediction tools are thought to be indispensable to anticipate the maximum number of patients requiring treatment.

From a clinical perspective, SARS-CoV-2 has an incubation period of 7 days, according to the World Health Organization (WHO) [3]. Other studies have reported an incubation period of 5-6 days [4-6]. Moreover, SARS-CoV-2 can be transmitted even before the onset of symptoms in infected individuals. We recognize the presence of infection acquired during the time it takes to carry out testing. Infections are not detected immediately after the infection but at a delayed timing because testing requires time. Hence, the date of true infection is some time before the date of detection. Therefore, real-time conditions cannot be known from future measurements. To take proper preventive action, a mathematical model is necessary to infer present conditions. The standard susceptible-infectious-removed (SIR) epidemic model [7] and most of its modified versions [8-10] have three compartments—the number of people who are susceptible, infectious, or have been removed either through recovery or death. Its derivative, the SEIR model [8], has another compartment, exposed (E) individuals, added to take a latency period into account. However, SARS-CoV-2 is different from most conventional infectious diseases in the point that unique symptoms are not well established yet and that patients with subclinical symptoms may be infectious [9]. Since an infectious patient cannot be identified clearly, contact between individuals in daily life needs to be limited, which creates substantial impact on social activities and the economy. Hence, it is important to locate and isolate infectious patients via testing, as isolation significantly affects the transmission. SIR/SEIR models have been standard tools used for this purpose [7,8].

SIR/SEIR models including a compartment for quarantined (*Q*) individuals are called SIQR [10] or SEIQR, respectively [11-13]. Some examples of derivatives include the inclusion of isolated patients in the SIR model [11], a delayed SEIQR epidemic model with a vaccination effect [12], or with quarantine and latent compartments [13]. The outbreak of SARS-CoV-2 has been analyzed by many authors in terms of quarantine rate [14-18]. Quarantine rate (ie, transition coefficient from compartment “infected” to “quarantined”) was estimated by available data under some simple assumptions [14] or using statistical methods [15], an AI (artificial intelligence) model [16], or a sophisticated 6-compartment model [17]. Cases in Japan was analyzed by Odagaki [18]. In the actual situation, however, the PCR (polymerase chain reaction) test followed by a quarantine action was executed at a later time after the infection. As a result, it has been surmised that the actual infection situation is reflected on the daily confirmed PCR test positive number by a delay of about 2 weeks in Japan [19]. Young et al [20] developed a delayed SEIQR model including this delay effect, which was applied to the COVID-19 context by Vyasarayani and Chatterjee [21]. All patients detected by PCR testing should be quarantined but their model does not always guarantee this due to its probabilistic approach.

The SIR model essentially suits analysis for a short-term epidemic in local districts [22]. It has been widely used mainly in developing countries in need of coping with various infectious diseases where the collective population is small [22]. However, this has changed in the context of COVID-19 as spread of infection is prevailing in developed countries with a large collective population.

The limitation of these SIR derivative models lie in the fact that in the case that the collective population is large (eg, Tokyo, Japan, or Wuhan, China), previous works solved only a part of the equation rather than the whole governing equation, with the assumption that susceptible individuals are replaced by the collective population (N) [15,18]. As a result, they construct an entire solution by connecting piecewise exponential function *exp*(λ*t*) with λ’s fitted by trend data corresponding to each piecewise period of the whole time domain. For example, Odagaki [18] fitted the trend of daily confirmed new cases in Japan in the March 1 to April 29 period with four piecewise exponential functions.

This paper attempts to propose a new epidemic model that provides not a combination of piecewise solutions but a direct simulation based on a discrete delay differential equation that includes the isolation period (hospitalization). This model is unique because of its inclusion of delay time *T* in the equation, and its ability to simulate a complete trend of various infectious variables from the beginning of the epidemic until the endpoint. We propose two models (PART1 and PART2). The former assumes that all infected cases lead to symptoms and eventually isolation, and was examined through various time-series data obtained from February 14 to May 23, 2020, in Tokyo [2]. The latter includes not only symptomatic but also asymptomatic cases (subclinical patients at large). Both models are capable of counting inpatient and infectious cases separately.

The relation between the fundamental reproduction number *R_0_* and the parameter of the present model is discussed. Furthermore, based on this knowledge, an exit strategy (a criterion for exiting the stay-at-home state of emergency) for the first wave [23] and how to cope with the coming second wave are discussed.

## Methods

### Data

For this study, we used a publicly available COVID-19 data set provided by the public health authority of the Tokyo Metropolitan Government in Japan [2]. The present epidemiological model was verified through various time-series data from February 28 to May 23, 2020, up to 2 days before the Japanese government declared a rescission of the state of emergency.

The average number of treatment days in hospital was estimated from data on the cumulative sum of discharge and deaths [2]. Simulations by the present model were examined by cumulative infections, daily new confirmed cases, detected and hospitalized, the number of inpatients, and recoveries/deaths in hospitals [2]. Numerical results were also examined via positivity ratio in PCR tests in Tokyo [2]. To establish the PART2 model, we used data from the report on antibody prevalence tests conducted by the Ministry of Health, Labor and Welfare from June 1 to 7, 2020, just after the end of the first wave in Tokyo [24]. These data were collected by public health authority announcements, were aggregate rather than individual case information, and were used only for the purpose of comparison with simulation results. Therefore, ethical approval was not considered to be required for this study.

### Prediction Models

#### An Epidemiological Model Considering Isolation With Delay

**Figure 1:**
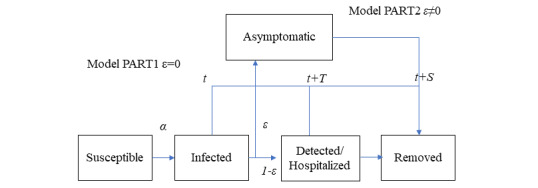
Concept of the present epidemic model. Two paths from newly infected until removed (recovered/died) are shown, with symptomatic and asymptomatic paths. The portion of the former is ε and the latter 1–ε. The former remains infectious in the time period [*t*, *t*+*T*] and the latter [*t*, *t+S*], with *T*<*S* (typically). We call the case ε=0 model PART1 and 0<ε<1 model PART2. The case ε=1 is equivalent to the standard SIR model, in which no distinction exists between symptomatic and asymptomatic cases.

Figure 1 displays the concept of the present epidemiological model. For simplicity, it is assumed that once the susceptible are infected at time *t* with a transmission rate of α (1/day), they become infectious without delay. Whether an infected individual becomes symptomatic or asymptomatic is assumed to be intrinsically determined. The asymptomatic cases remain infectious until removed (recovery/death) at time *t*+*S* while the symptomatic cases continue being infectious until hospitalization (isolation) at time *t*+*T*. Parameters *S* and *T* are not fitting parameters but are to be determined by empirical knowledge based on observed data. A part of infections (ε) is designated as asymptomatic (subclinical patients) and the rest (1–ε) as symptomatic. Taking cumulative infections *x*(*t*) as a primary dependent variable, then the number of infectious cases at large (*Q*) can be counted as a sum of asymptomatic portion ε(x(*t*) – *x*(*t*–*S*)) and symptomatic one (1–ε)(*x*(*t*) – *x*(*t*–*T*)). The susceptible portion in a collective population (*M*) can be expressed by 1 – *x*(*t*)/*M*. We assume the rate of infections (daily new cases) is proportional to the product of *Q* and the susceptible portion, that is, *dx*(*t*)/*dt* = α*Q*(1–*x*(*t*)/*M*). Then, *x*(*t*) is governed by the following delay differential equation:

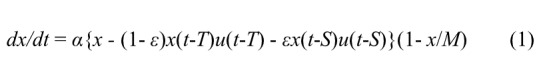



where *x*(*t*) is abbreviated by *x*, and *u*(*t*–*T*) or *u*(*t*–*S*) is a step function such that:

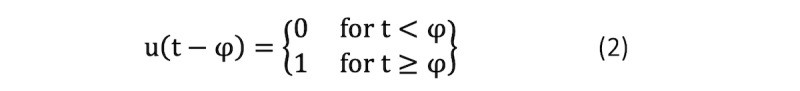



Given the number of initial infectors *x*(0) imported from outside of the collective population, parameters *S* and *T*, and a single set of fitting parameters α and *M* for an entire period of epidemic, a numerical solution would be obtained.

Equation 1 is featured in an explicit inclusion of time lag parameters, *S* and *T*, which is different from the SIR model and its derivatives. We designate this formulation as the apparent time-lag model (ATLM) hereafter.

The model for ε=0 treats clinical symptoms alone and does not take asymptomatic infections (subclinical patients) into consideration. Hence, all infections are to be eventually detected and hospitalized, and equation 1 becomes equation 3, which we refer to as the epidemic model PART1:

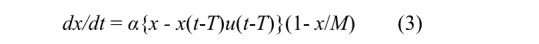



In the case ε=1, an alternative formulation equivalent to a standard SIR model is obtained. In the case 0<ε<1, we call equation 1 the epidemic model PART2 (see Multimedia Appendix 1 for details). For the numerical integration of equations 1-3, the fourth-order Runge-Kutta-Gill method was applied. With a time step of half a day, numerical accuracy was found to be adequate.

In the present model, the whole epidemic trend was simulated by a single delay differential equation in terms of cumulative infections *x* as the dependent variable.

Once *x* is obtained, other important variables would be evaluated in a straightforward way. For example, daily new cases *dx/dt* can be calculated by the right-hand side of equation 3, the number of hospitalized *x*(*t*–*T*) designated by *Y*, removed (recovered/died) *x*(*t*–*S*) by *Z*, infectious cases at large *x*–*Y* by *Q*, the number of inpatients *Y*–*Z* by *P* and PCR test positivity ratio *Q*/*M* by *Pr*.

In the subsequent section, we will focus our attention on model PART1. Table 1 summarizes the variables and their corresponding expressions.

**Table 1:** Expressions for infection variables in terms of cumulative infections x.

Item	Variable	Expression
Cumulative onset	*x*	—^a^
Hospitalized	*Y*	*x*(*t*–*T*)
Recovered/died	*Z*	*x*(*t*–*S*)
Inpatients	*P*	*Y*–*Z*
Infectious cases in the field	*Q*	*x*–*Y*
Positivity ratio for PCR^b^ testing	*Pr*	*Q*/*M* × 100 (%)

^a^Not applicable.

^b^PCR: polymerase chain reaction.

The value of time interval *T* was inferred as the sum of the incubation time, detection, and testing and reporting times. As mentioned already, the WHO announced that the incubation time of SARS-CoV-2 to be 7 days [3]; in other studies in the literature, it was reported as 5-6 days [4-6]. In actuality, however, the PCR test followed by a quarantine action was executed at a later time after the infection. As a result, the actual infection situation has been said to reflect in the daily confirmed PCR test positive number after a delay of about 2 weeks in Japan, in which the time from specimen collection to reporting back to the patient is delayed. Assuming an incubation period of 5 days [25], an infectious period of presymptomatic cases of 2 days [3], and a reporting delay of PCR test results of 3 days [26], infectious patients might not be quarantined until about 14 days. Hence, in the analysis of the first wave of the SARS-CoV-2 epidemic in Tokyo, we assumed *T* to be 14 days.

### Dimensionless Parameter and the Final Size of the Epidemic

Equation 3 can be simplified to respective time spans for 0<*t*<T as follows:

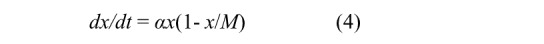



This equation has an analytic solution, a so-called logistic function:

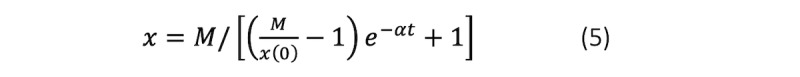



At the beginning, equation 5 shows an exponential epidemic growth *x = x(0)e^αt^ = x*(0)*2*^(^*^t/τ^* ^)^, where τ is clinical doubling time and related to α as shown below:

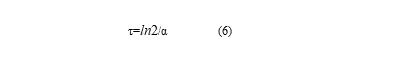



For example, with α being 0.164, the equivalent value of τ would be 4.22 days; x/x(0) would become 10 in 2 weeks.

When *t*>*T*,

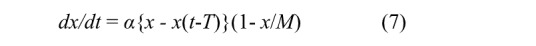



Normalization of equation 7 provides the following:

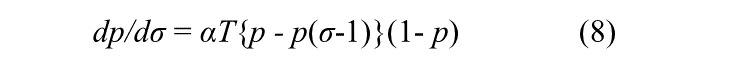



where *p*=*x*/*M* and σ=*t*/*T*. It should be noted that a single dimensionless parameter (α*T*) appears in equation 8 and governs epidemic behavior.

Here, we have derived a final size equation from equation 7:

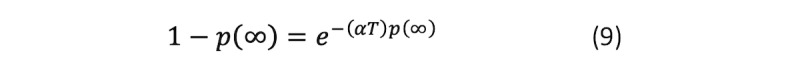



Solving for final size *x*(∞) is obtained as *Mp*(∞). Mathematical proof of equation 9 is available in Multimedia Appendix 2.

It is interesting to note that once α*T* is known in the early phase of the epidemic, the final size is also known without a numerical analysis. In other words, if we happen to know the final size *x*(∞) as well as α*T* in advance, we can estimate the virtual collective population *M*.

### Effective Reproductive Number

The effective reproductive number *R_t_* refers to the number of infections per infectious cases in a collective population until removal. Various calculation methods have been reported [27-29] for *R_0_* but less for *R_t_*. Its expression is dependent on the epidemiological model. Hattaf [27] derived *R_0_* for delayed SEIR model and showed delay reduces *R_0_*. Wallinga and Lipsitch [28] showed a framework for deriving *R_0_*. In the present model, possible infectors at time *t* exist within a time span [*t*–*T*, *t*] whose number is *Q* because the infectors produced before *t*–*T* are all hospitalized. Consequently, for this paper, *R_t_* was calculated as the ratio of daily new cases to the average number of infectants per day during time span [*t*–*T*, *t*] as:

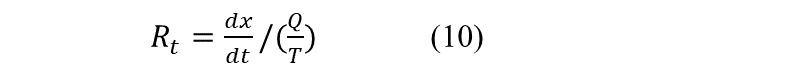



Combining equation 10 with equation 7 yields:

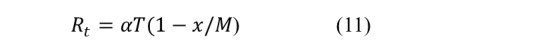



This is consistent with the literature [17]; the effective (time-dependent) reproductive number *R_t_* is the product of *R_0_* and susceptible *S*(*t*) if α*T* is the same as *R_0_*.

Since no preventive measure was taken at an initial stage of the first wave of the COVID-19 pandemic from February 14 until May 23 in Tokyo, the value of *R_t_* at the initial phase coincides with that of the basic reproductive number *R_0_*. Hence, noting 1>*x*(0)/*M*, we have:

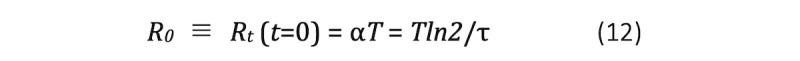



Now that dimensionless parameter α*T* has been identified as the basic reproductive number *R_0_* in the present model, *R_t_* in equation 11 is the effective reproductive number. Furthermore, the physical meaning of *R_0_* was clarified, which is the product of the ratio of time lag *T* and epidemic doubling time multiplied by *ln2*. In contrast, Makino [30] and Inaba [22] reported that the standard SIR epidemic model provides β*N/γ* for the basic reproductive number:

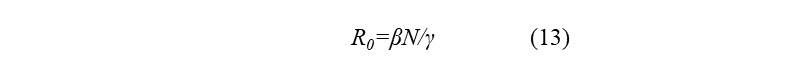



Recalling β*N*=α and 1/*γ* as the time constant, equation 13 has a meaning similar to equation 12.

## Results

### A Dimensionless Parameter to Characterize Early Stage Epidemic Trends

**Figure 2:**
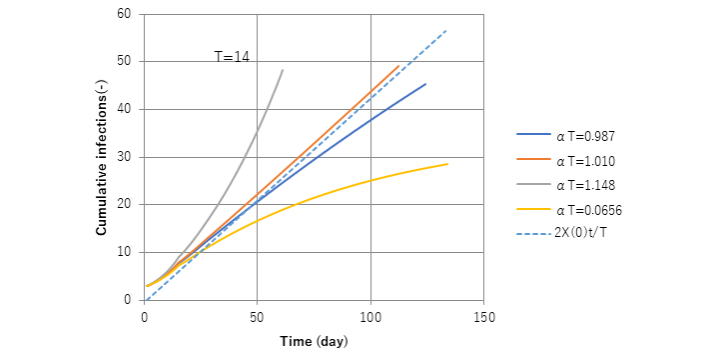
Behavior of solution x with a change in the value of the parameter (α*T*). A clear change in the behavior of the solution x(t) depending on the value of α*T*, a so-called bifurcation nature, is apparent. In fact, α*T*=1 is found to be a threshold with α*T*>1 causing an outbreak and α*T*<1 an endemic. This is consistent with the interpretation that α*T* is equal to *R*0 in equation 12.

Figure 2 presents an early stage epidemic trend for the solution *x*(*t*) of equation 7. The dimensionless parameter (α*T*) is equivalent to *R_0_* in equation 12. To confirm whether it is correct or not, a numerical calculation was conducted with (α*T*) as a parameter. It is clear in the case (α*T*)>1, cumulative cases *x* show exponential growth of the epidemic; when (α*T*)<1, they seem to be saturated. The marginal line is expressed as a broken line in Figure 2, when (α*T*)=1. With these features, (α*T*) is identified to be *R_0_*.

### Comparison of PART1 Prediction With Observed Trend Data

Cumulative confirmed cases between February 28 and April 28 in the first wave of COVID-19 in Tokyo were adopted for the fitting of parameters, transmission rate α, and virtual collective population *M*. Values of other parameters, time lags from infection until isolation *T* and from infection until removed *S*, were preset as 14 and 36 based on empirical knowledge. The former has been commonly acknowledged for SARS-CoV-2 in Japan. The latter was derived as a sum of *T* and average length of stay (LOS) in hospital until discharge or death. The average LOS days of inpatients was estimated to be 22 days based on the data for a total sum of discharge and deaths. Model PART1 was used. According to parameter survey calculations, α and *M* were selected, respectively, as 0.164 and 6200. In the following sections, the number 0.164 will be designated as α0 and used throughout the text in later sections.

**Figure 3:**
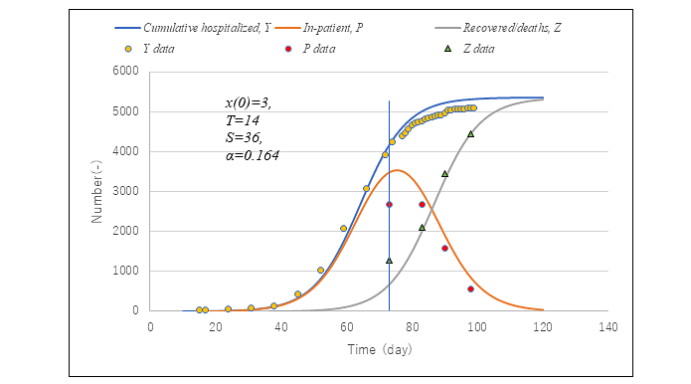
Comparison of simulation results with data in the entire period of the first wave of COVID-19 in Tokyo. Axis represents the number of cumulative hospitalized cases (Y), the number of inpatients (P), and the number of recovered/deaths (Z). Solid lines represent simulations by model PART1. Dotted points show data observed in Tokyo, Japan.

Figure 3 presents a comparison of epidemic simulation against observed data in terms of the cumulative hospitalization number (*Y*), the number of inpatients (*P*), and removed cases (recovered/died) (*Z*) in hospitals. Whereas observed data are available for February 28 onward, calculations started with initial infection *x*(0)=3 on February 14, which was 14 days prior. The vertical line indicates the date April 28. Calculations succeeded in simulating observed data in general. However, *Y* starts overestimating data at 77 days (May 2) and afterward. It is probable that α became smaller due to stay-at-home orders announced by metropolitan authorities 5 days earlier on April 27, by which point people’s behavior changes lowered the value of transmission rate α.

**Figure 4:**
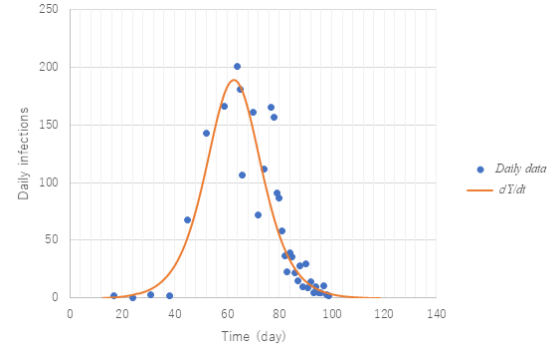
Simulation of daily new confirmed cases compared with observed data. Model PART1 with the same parameter values as in Figure 3 was used. Note that *dY/dt*=*dx*(*t*–*T*)/*dt* since nearly 2 weeks are needed in Japan to confirm infection by polymerase chain reaction testing.

**Figure 5:**
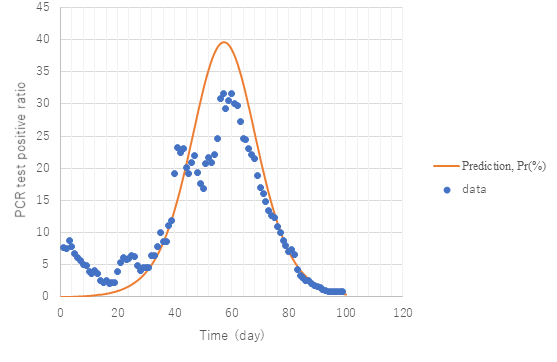
Simulation of positivity ratio compared with observed PCR (polymerase chain reaction) tests. Model PART1 with the same parameter values as in Figure 3 was used for the simulation.

Figure 4 presents a comparison of predictions with observed data on daily new confirmed cases from February 28 to May 23, that is, the whole span of the first wave in Tokyo. Although the data show remarkable scattering, prediction succeeded in simulating their average trend at large especially toward the end of the epidemic. It should be noted that during the first wave of the epidemic in Tokyo, the Metropolitan Tokyo Government asked those with a positive PCR test to stay in hospital until they were confirmed negative again twice. Therefore, PCR positives are equated with those who were hospitalized. Figure 5 exhibits observed trends of the positivity ratio in PCR testing compared with calculation *Pr* (equation 13).

Data in the early stage might include large statistical error because of the fewer inspections conducted. Except for this period, however, data trends are well simulated in spite of the model simplicity. That is reasonable because the model counted infections with the onset of clinical symptoms, which suits the attribute of tested data. It is important to note that the agreement of numerical results with observed data implies correctness of counting infectious cases at large (*Q*) because of the definition of the PCR positive ratio, *Pr*≡*Q*/M*100 (%), in the present model. Accuracy is recognized toward the endpoint of the epidemic.

### Trends in the Effective Reproductive Number

**Figure 6:**
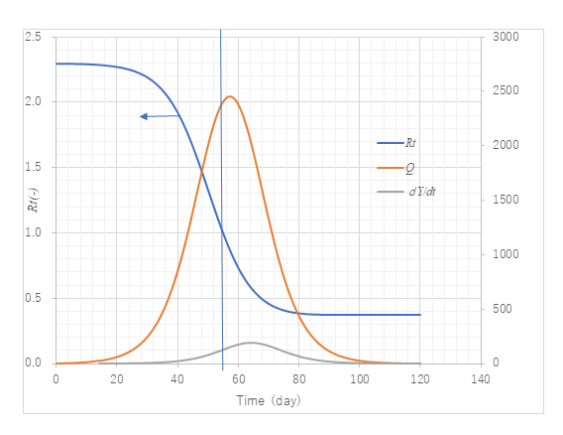
Trends of the reproductive number, *R_t_*, infectious cases at large, and daily new confirmed cases. Model PART1 with the same parameter values as in Figure 3 was used. The value of *R_t_* is on the left axis and the other variables, infectious cases at large (Q) and daily new confirmed cases (dY/dt), are on the right axis. The vertical line represents the date when *R_t_*=1, which almost coincides with the date maximum Q is reached. This verifies the expression of *R_t_*.

Figure 6 provides trends of infectious cases at large, daily new confirmed cases, and *R_t_*. Different from the method of past literature, *R_t_* in the present model is expressed by a continuous convex function. Infectious cases at large were predicted to have a peak of 2363 on day 57 (April 12), whereas the effective reproductive number *R_t_* decreases to reach unity on day 54 (April 9). The vertical line indicates April 9 when it crosses the point where *R_t_*=1. Both dates are close and reasonable. Susceptible persons remain uninfected by about 1000 susceptible individuals who are not infected. The value of α*T* (=*R_0_*) is estimated as 2.30 using equation 11 with the transmission rate α as α0. The value of α0, however, is influenced by the Japanese government’s declaration of emergency issued on April 7, which might underestimate the value at the initial stage of the epidemic. Therefore, the actual *R_0_* might be higher.

The vaccine ratio needed to avoid an outbreak in Tokyo was estimated using equation 11. To calculate the condition *R_t_*<1 at *t*=0 when the epidemic fades out, the following equation can be used:

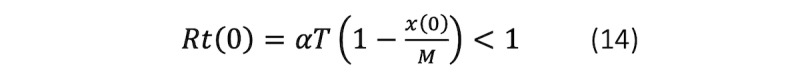



Rearrangement provides the vaccine ratio as:

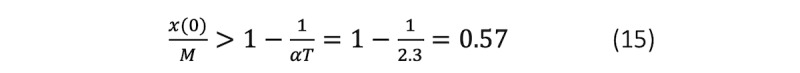



This estimate is reasonable since it is close to the value of 0.63 that Wu et al [31] obtained for the COVID-19 epidemic in Wuhan, China, as of January 23, 2020.

### Comparison of ATLM With SIR

**Figure 7:**
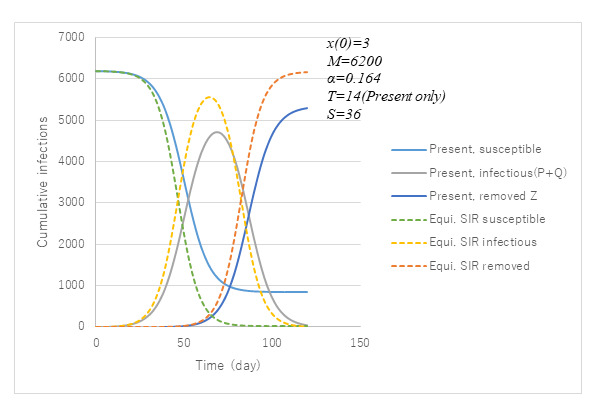
Comparison of simulations by the present apparent time-lag model (ATLM) with that of the standard susceptible-infectious-removed (SIR) equivalent. The results obtainable by SIR are based on equation 1 with ε=1 shown as a broken line and those by ATLM model PART1 as a solid line. Variables for infectious, removed and susceptible in SIR model correspond to those P+Q, Z and M-x, respectively in ATLM model PART1.

Figure 7 presents a comparison of ATLM with standard SIR epidemiological models in terms of 
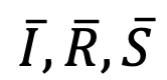
 under the same parameter values. Their corresponding variables in ATLM are, respectively, *P*+*Q*, *Z*, and *M–Z*. Marked differences are apparent between the two. In fact, SIR predicted that most of the population would be infected, although ATLM left behind 1000 as uninfected. This is because the attack rate *p*(∞) of ATLM is 0.86, which is smaller than that of SIR (almost 1) since α*T*<α*S*. Furthermore, ATLM provides lower values by 20% for infections, which results from modeling hospitalizations before removal.

### Results by PART2

According to the report of antibody prevalence tests conducted by the Ministry of Health, Labor and Welfare from June 1-7, 2020, just after the end of the first wave in Tokyo, the antibody ratio was found to be 0.10%. Since the metropolitan population is 14 million people, the number of individuals having antibodies is estimated to be 14,000. As the number of removed cases in the first wave was 5236 as of May 3 in Tokyo, this leaves 9000 in the field. Taking ε as 9000/14,200=0.634 as the first estimate, simulation of the first wave was conducted using PART2 to reproduce 5200 for (1–ε)*x*(∞) with ε varied. The best fit value of ε was 0.627.

**Figure 8:**
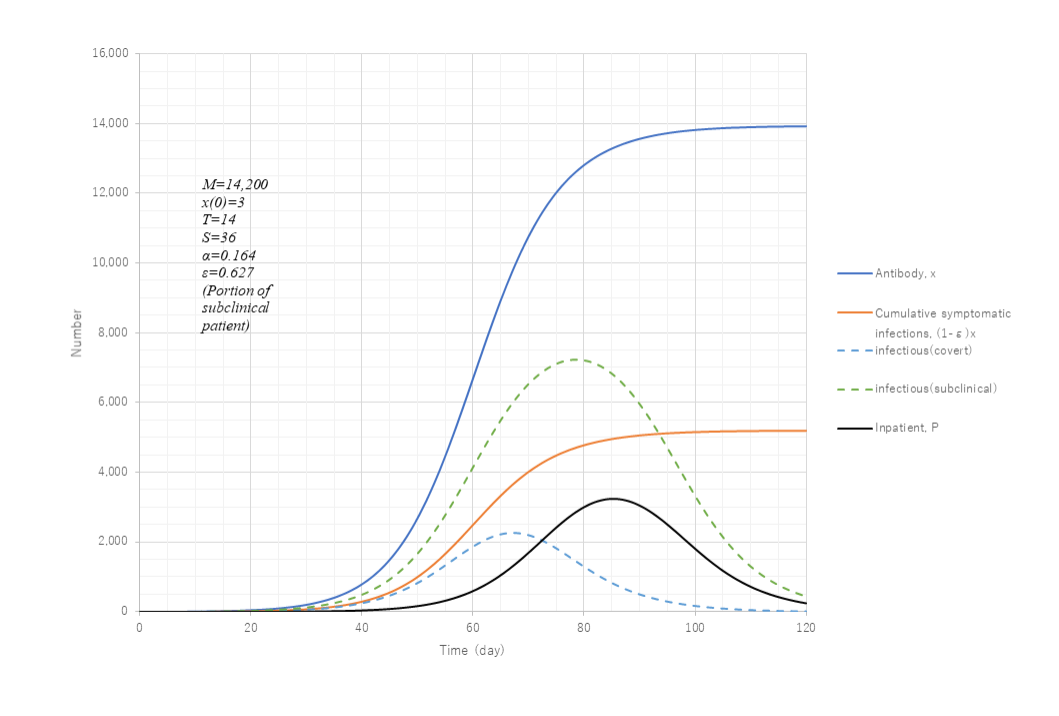
Model calculation with subclinical patients considered. Model PART2 (equation 1) was used. Model parameter M was determined to be 14,200 with the aid of the antibody prevalence test results. Parameter ε was optimized so as to give cumulative symptomatic infections, (1-ε)x is the same as that computed by PART1. The remaining parameter values of x(0), *T*, *S*, and α were the same as PART1. Broken lines represent infectious cases as covert and subclinical separately.

Figure 8 presents the trends of various variables with asymptomatic cases considered. Compared with *Q* of PART1 in Figure 3, the peak value of the apparent spreader (ie, the symptomatic or covert patient) inferred from PART2 is 90% in size with an inpatient ratio of 92%, both of which seem to be reasonable. Apparent to asymptomatic patients, the ratio is 0.45, which is less than the relative existence ratio (1–ε)/ε=0.6. This may be due to a difference in the values of *T* and *S*. As *S*/*T* is 2.6, a silent spreader continues to reproduce infections after an apparent spreader is isolated. Further information on incubation as well as the recovery period of silent spreaders are needed for improved accuracy.

### Assessment of Preventive Measures Against Spread

**Figure 9:**
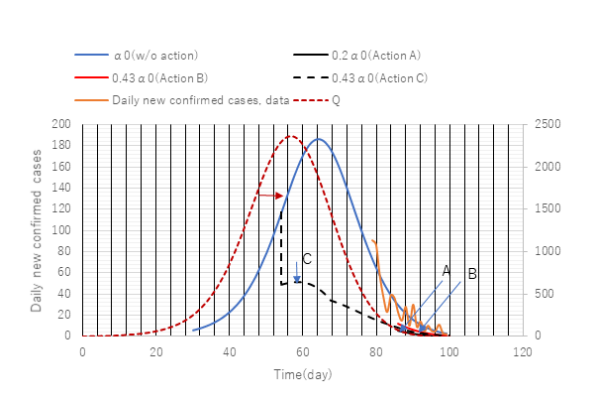
A posteriori assessment of the impact of restrained contact to reduce transmission rate, α. Stepwise change in the value of transmission rate α from α0 to 0.2α0 (action A) or to 0.43α0 (action B) was applied at day 86 when the effect of the action should appear on daily new confirmed cases. Solid lines represent daily new confirmed cases (left axis) while the red broken line (infectious cases at large) to the right. It was found that “real” action A was too late in suppressing spread. If “imaginary” action C had been implemented 1 month earlier, it would have had a greater impact.

Toward the end of the first wave in Tokyo, strong public health interventions were conducted to prevent contact with others by 80% (stay-at-home orders issued for 80% of residents). The actual reduction in the number of contacts was estimated to be 50%-60%. The official stay-at-home announcement was declared on day 72 (April 27) after the onset of the epidemic. The simulation was tailored to assess its effects on daily new confirmed cases with and without stay-at-home actions. Results are presented in Figure 9.

In Figure 9, actions A, B, and C are assumed public preventive measures against the spread of SARS-CoV-2. The government requested a reduction of contact between persons by 80%. In the simulation, this was modeled by a sudden decrease in the transmission rate. In a general form of φα0, φ ranges as follows: 0<φ<1. For instance, 0.2α0 implies an 80% reduction of contact compared with no action (φ=1).

In Figure 9, simulation of daily new confirmed cases with three transmission rates of α0 (no action), 0.43α0 (action B), and 0.2α0 (action A) were given together with PCR test data (orange color), with an α0 value of 0.164. Unexpectedly, the measured data appear to follow the case with no action *dY/dt*, which implies that the action failed. According to the postanalysis by ATLM, the action should have been conducted earlier. Imaginary action with 0.43α0 (action C) was taken at day 40. Its effect appears on daily new cases data 2 weeks later (day 54) when computational action was made. A significant effect on a reduction in infections and a reduced impact on social and economic activities might have been obtained.

### Assessment of Criterion for Rescission of Emergency Statement

**Figure 10:**
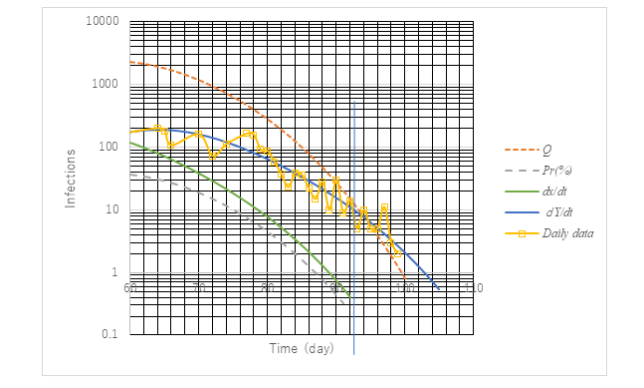
Trends of infectious variables as SARS-CoV-2 transmission declines. *dx/dt* represents a simulation of the actual trend of daily new cases. *dY/dt* was drawn with the curve *dx/dt* shifted to the right by 2 weeks. The daily data show daily new confirmed cases based on a positive polymerase chain reaction test. Both agreed well. The vertical blue line exhibits the date of rescission of the state of emergency declared by the Government of Japan. Then, the rescission criterion, that is, no more than 10 infections per day in terms of *dY/dt*, is satisfied. Infectious cases at large (Q), however, was estimated to have been 10 then according to the simulation. It should have been less than unity to aim for the extinction of SARS-CoV-2. To realize this, the rescission must be delayed by an additional week.

The Japanese government issued a criterion for the rescission of the stay-at-home order for the purpose of early economic recovery, stipulating that daily infections decrease to no more than 0.5 person/day per 100,000 population. Applied to Tokyo, the criterion would yield 10 infections per day. To confirm its validity, a posterior assessment was conducted. Figure 10 presents trends of infectious variables toward the end of the first wave in Tokyo.

The criterion of 10 infections/day can be represented as *dY/dt* (broken line in Figure 10) at day 93 after the onset of the first wave (February 14). Noting that measured values appear with a 14-day delay, the actual number is predicted to be 0.3, according to *dx/dt*. This number is below unity and is low enough to meet the rescission criterion. Infectious cases at large (infectious people who are not hospitalized), however, are still around 10 according to the simulation by ATLM (red broken line). Hence, the rescission should have been delayed by an additional 7 days (100 days afterward).

### Parametric Effect of the Public Intervention Against the Coming Wave

**Figure 11:**
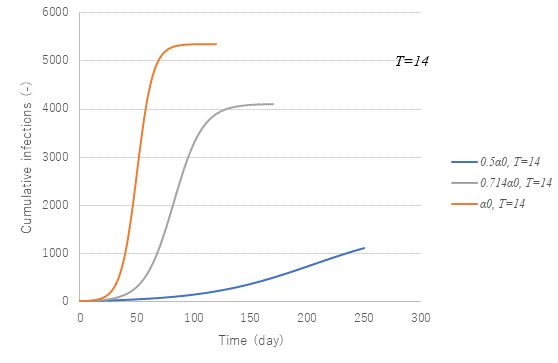
Effect of transmission rate α on cumulative infections. With a reduced transmission rate α, the final size x(∞) was observed to be smaller and took longer to be attained.

**Figure 12:**
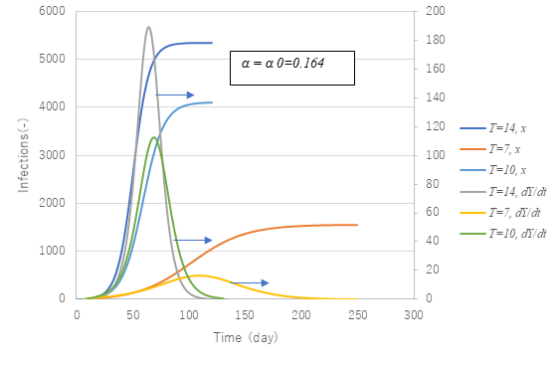
Effect of time lag *T* from infection until hospitalization on cumulative infections x (left axis) and daily new confirmed cases *dY/dt* (right axis). The reduction of *T* has a significant effect on both x and *dY/dt*.

Figures 11 and 12 show parametric effects. Calculations with parameter set of *t*=14 and α0 are set for the first wave and standard case. A smaller α is seen to reduce the second wave markedly.

A similar effect is expected by reduction of time lag *T* for both cumulative and daily new confirmed cases. Recovery of social and economic activities must accompany infections in a tolerable range. Consequently, a reduction in *T* under an increase in α is expected to be the basic policy for the coming wave followed by the first wave.

**Figure 13:**
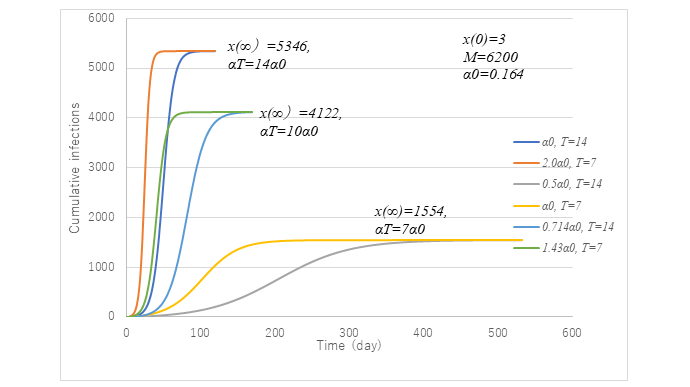
Effect of α*T* on x(∞). Model PART1 was applied with parameter values same as those in Figure 3 except for transmission rate α and time lag *T*. Given their product α*T*, the final size x(∞) could be uniquely determined.

Figure 13 presents trends of cumulative cases with three (α*T*) values at 14α0. 10α0 and 7α0 have two curves each. Among them, the parameter set of *t*=14 and α0 is the standard case used to simulate the first wave. It is noteworthy that the asymptotic values *x*(∞) of the two curves under the common α*T* are the same although initial increasing rates are different. It can be seen that the lower the (α*T*), the smaller the magnitude of the epidemic. From this observation, three sets of parameter combinations (α*T*, *x*(∞)/*M*)—(1.148, 0.251), (1.640, 0.665), and (2.296, 0.862)—were obtained.

**Figure 14:**
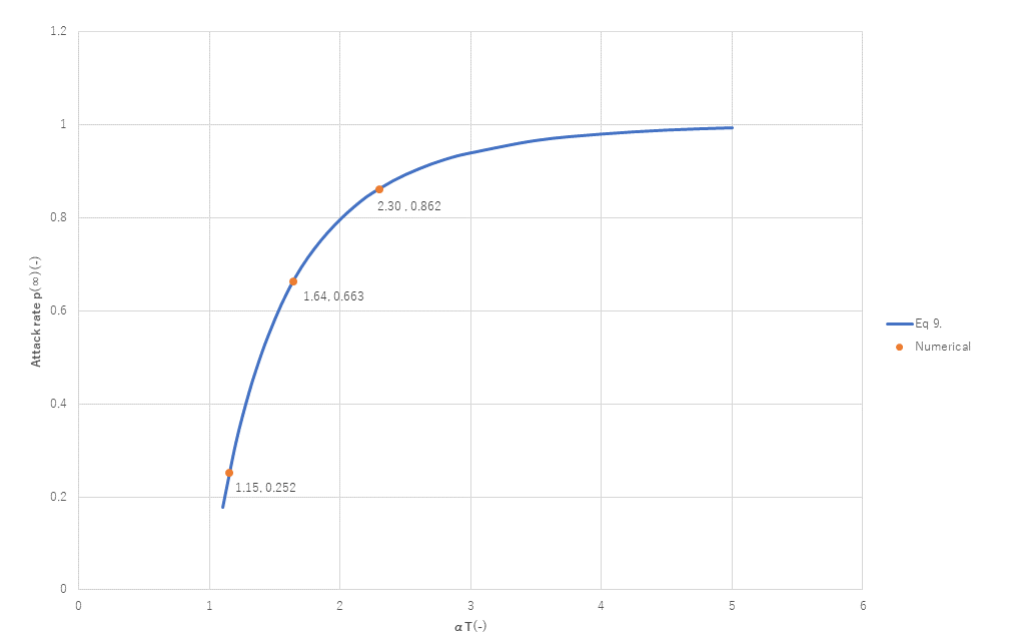
Relationship between attack rate p(∞) and α*T*. The accuracy of equation 9 is verified by this numerical simulation.

Figure 14 presents the accuracy of equation 9. Excellent agreement with numerical results is obtained. In the region (α*T*) <3 strong correlation are observed between *p*(∞) and (α*T*), but the attack rate tends to become saturated to unity if (α*T*) exceeds 3.

**Figure 15:**
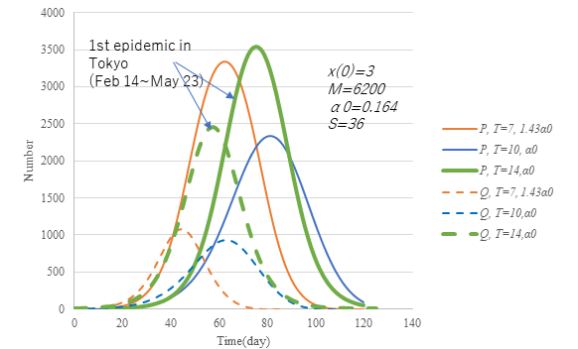
Effect of *T* on the number of inpatient and infectious cases at large under the condition of (α*T*)=10α0. Model PART1 was applied with parameter values same as in Figure 3 except transmission rate α and time lag *T*. Solid lines represent the number of inpatients (P) and broken lines the number of infectious at large (Q). The reduction of *T* and an increase in α may provide a solution that reduces inpatient count and enhances economic activity.

Another point of checking is to prepare the necessary number of hospital beds. Figure 15 exhibits the number of inpatients and infectious cases at large under α*t*=10α0 (ie, attack rate of 0.665). Two cases are compared with the case of the first wave in terms of beds and infectious individuals at large. The peak number of infectious cases is seen to be reduced to half the size of the first wave for both cases. In the case of *T*=7 with 1.43α0, the epidemic will cease 1 month earlier although the maximum number of beds is much the same as that in the first wave. Unless *T*=7 is feasible, the next choice would be *t*=10 with α0, which would require a reduced number of beds with a delayed transmission endpoint. From an economic point of view, *T*=7 is preferable because α is bigger. Earlier identification of infectious cases at large is essential.

## Discussion

### Principal Findings

The present epidemiological model ATLM is very simple in terms of mathematics and comprises a small number of fitting parameters, that is, the governing equation is described by only one dependent variable *x* (cumulative infections). Once it is solved numerically, other infection-related variables can be obtained in a straightforward manner as a function of *x*, as illustrated in Table 1. On the other hand, conventional approaches are complex. For example, the mathematics of SIR and its derivatives consists of multiple equations with variables depending on the number of compartments of community in the model—SEIR [8] or SIQR [10] uses four compartments, SEIQR [11-13] five, and delayed SEIQR [20] six. In essence, they have not been solved directly in the entire time span but have been approximated by a combination of piecewise exponential functions, each of which is a solution applicable to a short-term interval with a fitting parameter. For example, Odagaki [18] divided the entire time span of the first epidemic in Japan (March 1 to April 30) into four intervals and applied an SIQR model with four parameter values fitted to each interval, that is, in total 16 (4×4), whereas ATLM employs only four parameters. The accuracy of ATLM was examined by various data obtained from the first SARS-CoV-2 trends [2]. They are cumulative infections *x*, daily new confirmed cases *dY*/*dt*, the number of inpatients *P*, discharge and deaths *Z*, and trend of PCR positivity ratio *Pr*. All of these are filed in the database [2] and were simulated well by ATLM PART1. It should be noted that this was done so by a single set of four parameters for a transmission rate α of 0.164, a virtual collective population *M* of 6200, a time lag *T* of 14 from infection until isolation or hospitalization, and a time interval *S* of 36. ATLM succeeded in simulating the whole data trend of the first wave of the COVID-19 epidemic in Tokyo.

Among them it is noteworthy that the simulation matched the trend of the positivity ratio of PCR testing. This implies that the number of infectious cases at large was counted properly by ATLM. This fact may essentially be a base to apply simulation results to the assessment or proposal of strategies for public control of the epidemic (ie, public health interventions at the right magnitude and timing). We demonstrate two examples below.

One is the assessment of the stay-at-home order to reduce person-to-person contact by 80% declared by the metropolitan authority on April 27 (72 days after the onset of the first wave of the epidemic). Based on the prediction of infectious cases at large by the present model, the declaration should have been made 1 month earlier. If so, moderate reduction of contact by 43% would have been effective enough to reduce both inpatient and impact on social and economic activities.

The second example is the timing of the rescission of the state of emergency issued on April 7. It was actually done on May 25 based on PCR test results. However, according to the behavior of the infectious cases at large, it should have been postponed by 1 week when the calculated infectious cases at large would reach below one at which point the epidemic would cease.

As a corollary, we induced a single dimensionless parameter (α*T*) from the governing equation that occupies the whole epidemic trend from onset until endpoint. More specifically, (α*T*) was identified as *R_0_*; it also determines attack rate *p*(∞) and the final size of the epidemic *x*(∞). In practice, once we know (α*T*) in the early phase by data fitting, we can estimate the attack rate of the specified epidemic without numerical simulation. The value of (α*T*) obtained was 2.30 for the first wave of the epidemic in Tokyo; this value is close to the value of 2.20 that Li et al [6] obtained from the data taken from Wuhan, China, or the value 2.68 reported by Wu et al [31] for SARS-CoV-2.

As for the time-dependent effective reproductive number *R_t_*, conventionally it was evaluated as a piecewise function [18]. However, ATLM expresses *R_t_* as a function of *x*(*t*) applicable to the entire period of epidemic. This is consistent with findings from the literature [17] that an effective (time-dependent) reproductive number *R_t_* is the product of *R_0_* and susceptible *S*(*t*).

Three parametric survey calculations for the preparation of a coming wave clarified the combination of *T*=7 and α=1.43α0 as providing the best solution for restoring social activity, with a smaller magnitude of cumulative infections. To make *T* smaller in practice, faster identification and quarantine of infectious cases at large are necessary. This requires a strong task force to find infection clusters and to apply rapid testing to the greatest degree possible.

### Limitations and Future Work

The present model (ATLM) has limitations. First, it is assumed that all new cases spread the infection from the time of infection *t* until *t*+*T* isolation in hospital. In fact, a noninfectious period (exposed period) exists at the nascent stage of incubation, which may reduce the infectious period. This mechanism could be formulated within the frame of ATLM by introducing another time lag for the exposed period. It is our future task to complete this with a reliable empirical database. To do so, more information on incubation as well as recovery period of asymptomatic patients are needed for accurate modeling.

After June 2020, PCR testing was enhanced in Japan in order to suppress infectious subclinical patients as a measure of intensive cluster intervention. As a result, the number of positive PCR tests increased compared with the first wave of the epidemic and a similar number of subclinical patients as inpatients was found. Nevertheless, the maximum number of hospital beds required were less than that of the first wave in Tokyo. This may be owing to improved medical care as well as the larger portion of young patients. Postanalysis of the epidemic from June to October 2020 using general ATLM PART2 (equation 1) with information mentioned above will be our next task.

Secondly, the characteristics of the onset of clinical symptoms are stochastic. Therefore, the modeling of a preonset period and a statistical process are needed for accurate prediction. Thirdly, we assumed in the model PART2 that subclinical patients continue to be infectious from infection to removal, which must overestimate the number of infectious cases. With more information on period *S* from infection until recovery for subclinical patients, modeling should be improved.

Fourthly, input parameter *M* (virtual collective population) introduced into ATLM as a fitting parameter enabled us to simulate the entire span of the SARS-CoV-2 trend from the beginning through to the endpoint of the epidemic in Tokyo with a single value to designate transmission rate α. However, this should be modeled in the future. In ATLM, both transmission rate α and virtual collective population *M* were simultaneously determined by data fitting to cumulative cases. α can be alternatively understood as the reciprocal of clinical doubling time, that is, the accelerating factor of the spread. On the other hand, *M* is understood as the initial susceptible, a decelerating factor for the spread if *M* is small. In fact, *M* was extremely small (~5000) compared to the actual population of Tokyo (14 million). This might be related to the mobility of individuals in daily life. Populations outside the flow of individuals are not susceptible. To define the invisible wall between the susceptible and the nonsusceptible would be the key to model *M*. This will be addressed in our future work.

In summary, this study is the first complete simulation of the first wave of the epidemic in terms of the trends associated with various SARS-CoV-2 infection parameters in Tokyo, Japan. Existing data and outbreak patterns in other countries may be better understood via the present model.

### Conclusion

A novel epidemiological model (ATLM) was developed using a single delayed differential equation with explicit inclusion of the time lag associated with the isolation of infectious cases. It provides a full simulation of the various infection variables in the entire span from onset to endpoint with a small number of calculation parameters. The model was verified by various epidemic trend data (including the PCR positivity ratio) published by the Tokyo Metropolitan Government. The validity of counting infectious cases at large was checked indirectly by the coincidence of data for the PCR positivity ratio. Based on this, two practical issues about public health control of SARS-CoV-2 surfaced. One of them is the mitigation of infections by reducing social contact, declared on April 27, 2020. Based on the trend of infectious cases at large predicted by ATLM PART1, this order should have been issued 1 month earlier, which would have led to less infection as well as a reduced slowdown of social activities. The other issue is the timing of the declaration of the rescission of the state of emergency, which was issued on April 7 and rescinded on May 25. However, according to the predicted behavior of the infectious cases at large, this should have been done 1 week later when infectious cases are at <1 and the epidemic would fade out. Finally, as a control measure for a coming second wave, the combination of *T*=7 and α=1.43α0 is recommended, which would result in enhanced social activities and a smaller magnitude of cumulative infections.

## Appendix

Multimedia Appendix 1 Derivation of the general delay differential equation: consideration of silent spreaders (model PART2).

Multimedia Appendix 2 Derivation of the final size equation.

## Abbreviations

ATLM: apparent time lag model
PCR: polymerase chain reaction
SEIR: susceptible, exposed, infectious, and removed
SIR: susceptible, infectious, and removed
SIQR: susceptible, infectious, quarantine, and removed
SEIQR: susceptible, exposed, infectious, quarantine, and removed
WHO: World Health Organization
