# Supplementary material for: An Epidemiological Model Considering Isolation to Predict COVID-19 Trends in Tokyo, Japan: Numerical Analysis
Source: JMIR Public Health Surveill. 2020 Dec 16;6(4):e23624. doi: 10.2196/23624 (PMC7746226; doi:10.2196/23624)
Supplement: Multimedia Appendix 1 [file publichealth_v6i4e23624_app1.docx]

***Appendix A***

A1 *Standard SIR Epidemiological Model and Its Alternative Formulation*

The standard SIR epidemiological model is formulated as

$\frac{d\bar{I}}{dt}=\beta\bar{I}\bar{S}-\gamma\bar{I},$ (1)

$\frac{d\bar{R}}{dt}=\gamma\bar{I},$ (2)

$N=\bar{S}+\bar{I}+\bar{R}$ , (3)

where variables $\bar{S}$, $\bar{I}$, and $\bar{R}$ respectively denote susceptible, infectious, and removed and *N* represents the collective population under consideration. Also, *β* and *γ* are, transmission rates, the parameters to be determined. Here, we introduce cumulative infections *x* as the primary unknown variable and rewrite Eqs.(1)-(3). A delayed differential equation can be obtained as follows. Substituting Eq. (2) into Eq. (1) and noting the relation $x=\bar{I}+\bar{R}$ Eq. (1) can be rewritten as

$\frac{dx}{dt}=\beta\bar{I}\bar{S}$ . (4)

Differential operator *d*/*dt* represents the first-order delay with time constant 1/γ; it is equivalent to substitute $\bar{R}=x(t-S)$ for Eq. (2) in which all infectious cases were assumed to be removed after a period of time *S*. Then, with $\bar{S}=N-x$ from Eq. (3), one finally obtains a newly found delay differential equation with a single unknown variable *x* equivalent to Eqs. (1)–(3) as

$\frac{dx}{dt}=\beta\left( x-\bar{R} \right)\left( N-x \right)=\beta N(x-x\left( t-S \right)u\left( t-S \right))(1-x/N)$ , (5)

where $u$ is the step function defined as

$u\left( t-S \right)=\left\{ \begin{aligned} 0 for t<S \\ 1 for t\geq S \end{aligned} \right\}$. (6)

Ichiyoshi derived Eq.(5) intuitively but did not solve it [25].

In this formulation, input parameter *S* can be ascertained from measurements. We set the value of *S* as 36days that represent the sum of time interval *T* and treatment days in hospital until discharge. The latter was estimated from official data of Tokyo Metropolitan Government [2].

Eqs.(5) and (6) coincide with a special case of the general equation Eq.(15) with ε=1.

A2 *Epidemiological Model Considering Isolation with delay (PART1)*

For simplicity, the following assumptions were made in PART1.

1. Those cases infected before *t*-*T* are all detected and isolated inside a ward which can be expressed as *x*(*t*-*T*), denoted by *Y*. Those infected in period [*t*-*T*, *t*] denoted by *Q* (= *x*-*Y* ) exist outside the ward.
2. We assumed *Q* are infectious from infected until hospitalized and contain no removed (recovered/deaths) because *T<S*. As removal happens solely in hospital, the recovered cases and deaths in hospitals are *Z*. In-patient denoted by *P* can be represented as *Y*-*Z*. Therefore, *P*+*Q* becomes *x*-*Z* that corresponds to $\bar{I}$ in the SIR model.
3. The spread of infections inside wards was not examined in the present model. Consequently, $\bar{I}$ in Eq. (4) was replaced by *Q* in the present model. Then, we have a governing delay differential equation for PART1 with a single unknown variable *x* for isolation considered epidemiological model with delay T as

$\frac{dx}{dt}=\beta Q\bar{S}$=$\alpha(x-x\left( t-T \right))u\left( t-T \right)(1-\frac{x}{M})$, (7)

where *α( = βM)* is the transmission rate. We will designate the above-described formulation as the Apparent Time Lag Model (ATLM) hereinafter. This coincides with a special case of the general equation Eq.(15) with ε=0.

Here, α depends on the personal behavior of individuals in daily life such as social distancing, washing hands, masking, public health intervention, and control. *M* represents potential susceptible people at the beginning of an epidemic. *M* is not a geographical population, but a virtual one. In fact, *M* is much smaller than the actual population of Tokyo because the daily action range of a normal person is limited where the possible number of people that a person would meet is not large. Therefore, *M* cannot be ascertained before calculation: instead, it is inferred from parameter fitting to measurement data. It is important to note the difference between *N* in SIR and its derivatives and *M* in the present model. N has been taken as actual population. For example, in the case of simulation by the present epidemic model for 1^st^ epidemic in Tokyo, best estimate of *M* was 6200 while population of Tokyo is about 14million. Then the ratio of both is 6200/14000000=0.0004 being extremely small. *M* can be said as the possible number of susceptible at the beginning of an epidemic.

Once Eq. (7) is solved, important infectious variables are derived in terms of *x*.

Hospitalized *Y*=*x*(*t*-*T*) (8)

Recovered/deaths *Z*=*x*(*t*-*S*) (9)

Number of in-patient *P*=*Y*-*Z*=*x*(*t-T*)-*x*(*t-S*) (10)

Infectious people in field *Q*=*x*-*Y*=*x*-*x*(*t*-*T*) (11)

Positivity-ratio in PCR test *Pr*=*Q*/*M* × 100(%) (12)

A3 *Derivation of general delay differential equation: Consideration of Silent Spreaders (PART2)*

We will extend the model above to accommodate silent spreaders to evaluate antibody production. Silent spreaders are subclinical patient, asymptomatic but infectious people. Therefore, no isolation was possible for them in Tokyo because little PCR tests were applied to subclinical patients during February 28th and May 23rd. For simplicity we assume they continue to be infectious from infected until recovered / deaths. Presumably, the percentage of antibodies of silent spreaders is known a priori. It is independent of the model parameters α, M, T or S. Total infectious cases in the field are presumed to be the sum of contributions from both silent spreaders and covert patient. The latter obeys Eq. (7) in Appendix A. Let ε be the share of silent spreaders, infectious term *Q* in Eq. (7) might be replaced by (1-ε) *Q* + ε (*x*-*Z*) . Consequently, we have an extended delay differential equation including silent spreaders for the model PART2 as

$\frac{dx}{dt}=\alpha\left\{ \left( 1-\epsilon\right)Q+\epsilon(x-Z) \right\}\left( 1-\frac{x}{M} \right)$ (13)

$Q=x-Y=x-x\left( t-T \right)$

$$Z=x(t-S)$$

$\therefore\frac{dx}{dt}=\alpha\left\{ \left( 1-\epsilon\right)\left( x-x\left( t-T \right)u(t-T) \right)+\epsilon(x-x\left( t-S \right)u\left( t-S \right)) \right\}\left( 1-\frac{x}{M} \right).$ (14)

Rearrangement yields to a generalized form of ATLM

$\frac{dx}{dt}=\alpha\left[ x-\left( 1-\varepsilon\right)x\left( t-T \right)u\left( t-T \right)-\varepsilon x\left( t-S \right)u\left( t-S \right) \right]\left( 1-\frac{x}{M} \right)$ (15)

Eq.(15) with ε=0 coincides with Eq.(7) in Appendix A where all infected are symptomatic cases that are detected and isolated. With ε=1 it is equivalent to the standard SIR model. It should be noted infectious of the SIR model has no distinction between symptomatic and asymptomatic infectious but gives a sum of both.
