# Supplementary material for: An Epidemiological Model Considering Isolation to Predict COVID-19 Trends in Tokyo, Japan: Numerical Analysis
Source: JMIR Public Health Surveill. 2020 Dec 16;6(4):e23624. doi: 10.2196/23624 (PMC7746226; doi:10.2196/23624)
Supplement: Multimedia Appendix 2 [file publichealth_v6i4e23624_app2.docx]

***Appendix B***

*Derivation of Final Size Equation*

Governing equation of ATLM for PART1:

$\frac{\mathrm{dx}}{\mathrm{dt}}=\alpha\left\{ x-x\left( t-T \right)u(t-T) \right\}\left( 1-\frac{x}{M} \right)$, (1)

*x*+$\bar{S}$=*M,*

$\bar{I}$= $x-x\left( t-T \right)u\left( t-T \right),$

where $\bar{S,}\bar{I}$ and *M* are susceptible, infectious and collective number of peoples.

Equation (1) can be rewritten as

$\frac{d\bar{S}}{dt}=-\frac{\alpha}{M}\bar{I}\bar{S}$ ,

$\int_{0}^{\infty} \frac{d\bar{S}}{\bar{S}}=-\frac{\alpha}{M}\int_{0}^{\infty} \bar{I}dt$ . (2)

Let Laplace transformation $\mathcal{L}\left\{ x\left( t \right) \right\}\equiv X\left( s \right)$, then $\mathcal{L}\left\{ x\left( t-T \right)u\left( t-T \right) \right\}=e^{-sT}X\left( s \right).$

Further expansion gives

$\mathcal{L}\left\{ \int_{0}^{t} x\left( t \right)dt \right\}=\frac{X\left( s \right)}{s}-\frac{\lim_{t\to0} \int_{0}^{t} x\left( t \right)dt}{s}$, where $\frac{\lim_{t\to0} \int_{0}^{t} x\left( t \right)dt}{s}=\frac{\lim_{t\to0} tx(0)}{s}=0. \therefore\mathcal{L}\left\{ \int_{0}^{t} x\left( t \right)dt \right\}=\frac{X\left( s \right)}{s}$ .

From definition $x\left( 0-T \right)u\left( 0-T \right)=0$, then

$\mathcal{L}\left\{ \int_{0}^{t} x\left( t-T \right)u(t-T)dt \right\}=\frac{e^{-sT}X\left( s \right)}{s}$,

$J\left( s \right)\equiv\mathcal{L}\left\{ \int_{0}^{t} x\left( t \right)-x\left( t-T \right)u\left( t-T \right)dt \right\}=\frac{\left( 1-e^{-sT} \right)}{s}X\left( s \right).$

From final value theorem

$\underset{t\to\infty}{\int_{0}^{\infty} \bar{I}dt=lim} \int_{0}^{t} \bar{I}dt$=$\lim_{s\to0} sJ\left( s \right)=\lim_{s\to0} \frac{\left( 1-e^{-sT} \right)}{s}\left\{ sX(s) \right\}$=$\lim_{s\to0} Te^{-sT}\lim_{s\to0} \left\{ sX\left( s \right) \right\}=Tx\left( \infty\right),$

R.H.S. of Eq. (2) =$-\alpha T\frac{x(\infty)}{M}=-\alpha Tp\left( \infty\right)$, (3)

L.H.S. of Eq. (2) = ln$\frac{S(\infty)}{S(0)}$= ln$\frac{M-x(\infty)}{M}=ln(1-p\left( \infty\right))$. (4)

where $p\left( \infty\right)$ is the attack rate and is defined by $p\left( \infty\right)\equiv\frac{x(\infty)}{M}$. From Eqs. (3) and (4), we have

$1-p\left( \infty\right)=e^{-\alpha Tp\left( \infty\right)}$ . (5)

Similarly, for PART2 model of ATLM we have

$1-p\left( \infty\right)=e^{-\alpha\left[ \left( 1-\varepsilon\right)T+\varepsilon S \right]p(\infty)}$ (6)
